# Dynamic changes of throat swabs RNA and serum antibodies for SARS-CoV-2 and their diagnostic performances in patients with COVID-19

**DOI:** 10.1080/22221751.2020.1810133

**Published:** 2020-09-17

**Authors:** Xueping Qiu, Yang Xiang, Jianbin Sun, Xinrui Wang, Guoqiang Chen, Xianqun Xu, Shengjun Liao, Na Yang, Siwei Li, Gang Yang, Yueting Tang, Junli Fan, Wen Xie, Hanning Hu, Yirong Li, Fang Zheng

**Affiliations:** a Center for Gene Diagnosis & Department of Laboratory Medicine, Zhongnan Hospital of Wuhan University, Wuhan, People’s Republic of China; b Wuhan Third Hospital-Tongren Hospital of Wuhan University, Wuhan, People’s Republic of China; c Hebi City Center for Disease Control and Prevention, Hebi, People’s Republic of China; d Huang Gang Central Hospital, Huang Gang, People’s Republic of China

**Keywords:** Antibody, SARS-CoV-2, COVID-19, RNA, throat swabs

## Abstract

Dynamic changes of RNA and antibodies in SARS-CoV-2 infected patients remain largely unknown, and influence factors for antibody production have not been fully clarified. In this study, consecutive throat swabs specimens (*n* = 1875) from 187 patients were collected to analyse the dynamic changes of RNA. Moreover, 162 serial serum samples from 31 patients were tested for seroconversion of IgM and IgG. Meanwhile, IgM and IgG were also detected in 409 COVID-19 patients and 389 controls. Additionally, the logistic regression analysis was executed to identify the possible influence factors for antibody production. The median positive conversion time for RNA was day 7 (IQR, 3–11), and the positive rate was highest in day 1–5 (74.59 %) and then gradually decreased. The median time of seroconversion for IgM and IgG were both day 12 (IQR, 10–15). The sensitivity and specificity for IgM (or IgG) was 87.04% and 96.92%, respectively. Multivariate logistic regression indicated that reduced lymphocytes and short positive conversion time for SARS-CoV-2 RNA were independent factors for negative results of IgM and IgG. In conclusion, RNA and antibodies should be combined for COVID-19 diagnosis, and delayed seroconversion was influenced by the decreased lymphocytes and short positive conversion time for RNA.

## Introduction

Since novel coronavirus pneumonia (COVID-19) outbreak in December 2019, the number of reported cases has surpassed 3,672,238 with over 254,045 deaths worldwide, as of May 8, 2020 [1]. The severe acute respiratory syndrome coronavirus 2 (SARS-CoV-2), a member of coronaviruses is known to cause COVID-19 with common colds and severe illnesses such as Acute Respiratory Distress Syndrome (ARDS) [2].

A prompt and accurate diagnosis on the infection of SARS-CoV-2 is the cornerstone of the endeavours to offer appropriate management for patients and prevent further spread of the virus. So far, real-time quantitative PCR-based viral RNA detection and serological antibody determination are both the way to confirm the diagnosis of SARS-CoV-2 infection [3,4]. However, RNA testing based on throat swabs brought out a certain risk of false negative [5]. Some cases that were strongly epidemiologically linked to SARS-CoV-2 exposure and with typical lung radiological findings, but their throat swabs for SARS-CoV-2 RNA remained negative [6]. The performance of real-time quantitative PCR (RT-qPCR) depends on many factors, including sample types, different disease stages and skills for sample collection [7]. Though pharyngeal virus shedding was very high and infectious virus was readily isolated during the first week after onset of symptom, the RNA testing results turned to be negative from day-14 [8]. Compared with RT-qPCR, antibodies determination is advantageous with biosecurity, short turn-around time and high repeatability due to the homogeneity of blood samples [9]. However, antibodies titres are largely depended on the time course of antibody responses after infection, and they could be negative in the window periods [9]. So, we executed the present study to profile the positive conversion of RNA and antibodies, evaluate the diagnostic performances of RNA testing and antibody determination, and recommend a laboratory diagnostic flow in different stages of COVID-19.

## Materials and methods

### Sample collection

A total of 475 RT-qPCR confirmed cases of COVID-19 individuals and 389 cases of controls (non-COVID-19 patients) were enrolled from four medical institutions in Hubei Province between January 20 2020 and March 12 2020. These medical institutions included Zhongnan Hospital of Wuhan University, Wuhan Third Hospital-Tongren Hospital of Wuhan University, Huang Gang Central Hospital and Hebi City Center for Disease Control and Prevention. All cases were adult (age≧18) and the pregnant women were excluded in this study. The diagnosis and clinical classification criteria of COVID-19 was based on the Guidelines for Diagnosis and Treatment of Novel Coronavirus Pneumonia (7th version) [3], released by National Health Commission of the People’s Republic of China. The clinical data was obtained from the electronic medical records of patients. The data included demographic information, major comorbidities, the date of symptom onset, clinical classification, SARS-CoV-2 RNA testing results and other clinical laboratory test results. Written informed consent was waived and the study was approved by the Medical Ethical Committee of Zhongnan Hospital of Wuhan University, Wuhan Third Hospital-Tongren Hospital of Wuhan University, Huang Gang Central Hospital and Hebi City Center for Disease Control and Prevention.

### SARS-CoV-2 RNAs detection

Throat swabs were collected from patients and soaked into the virus preservation solution immediately (Zhongzhi, Wuhan, China). The total RNAs was extracted using the respiratory sample RNA isolation kit (Zhongzhi, Wuhan, China) according to the manufacture’s recommendation, and then amplified using reverse transcription real-time quantitative PCR assays (RT-qPCR), following the recommended standard operating protocols provided by the SARS-CoV-2 Nucleic Acid Diagnostic Kit (Sun Yat sen University Daan gene Co., Ltd, Guanzhou, China). The reaction mixture was used for both RNA reverse transcription and target genes amplification. The target genes include the open reading frame 1ab (ORF1ab) gene, and the nucleocapsid protein (N) gene of SARS-CoV-2. The results of the RT-qPCR were analysed according to the manufacturer’s protocol.

### Antibody testing

The IgM and IgG antibody against SARS-CoV-2 in serum samples were tested using chemiluminescent microparticle immunoassay (CLIA Microparticles) kits supplied by Autobio Diagnostics Co., Ltd (Henan, China), according to the manufacturer’s instructions. Briefly, the IgM μ-chain capture method was used to detect the IgM antibodies, based on the HRP-conjugated recombinant spike protein of SARS-CoV-2. In the first step, the IgM antibodies in samples bind to the mouse anti-human IgM coated on the microparticles. In the second step, after washing, the HPR-conjugated SARS-CoV-2 antigen in the Enzyme Conjugate was allowed to react with the SARS-CoV-2 IgM already bound to the solid phase in the first step. A complex of antibodies in the sample combined with enzyme-linked antigens was generated among the solid phase, by immunological reactions. However, the IgG antibodies were tested using an indirect method based on a recombinant spike antigen and HRP-conjugated anti-human IgG. In the first step, SARS-CoV-2 IgG antibodies in samples bind to recombinant SARS-CoV-2 antigen coated microparticles. In the second step, after washing, a complex of the SARS-CoV-2 IgG in samples binding with HRP-conjugated anti-human IgG were generated by immunological reactions, was fixed on the solid phase. In the following steps of IgM or IgG detection methods, chemiluminescent substrate was added, and then the complex catalyses substrate, resulting in a chemiluminescent reaction. The resulting chemiluminescent reaction was measured as RLUs (relative light units). The RLU was proportional to the amount of SARS-CoV-2 IgM/IgG in the samples.

The antibody levels were expressed using the relative binding signals compared to the cutoff value of each assay (S/CO). Samples with S/CO ≧ 1 were considered positive.

### Statistical analysis

Continuous variables were properly presented as mean ± SD or as median (IQR: interquartile range, IQR). Categorical variables were presented as frequencies (*n*) and percentages (%). Comparisons between continuous variables in different groups were performed by student’s *t* test or Mann–Whitney *U* test where necessary. Chi-squared test was used to compare categorical variables between groups, while the Fisher exact test was used when the data were limited. Univariate and multivariate binary logistic regression analysis were used to identify the influence factors for antibody testing results. The statistical analyses were conducted with the SPSS 20.0, all *p*-values were two-sided and *p* < 0.05 was considered statistically significant.

## Results

### SARS-CoV-2 RNA detection by RT-qPCR in consecutive throat swabs specimens for COVID-19 patients

A total of 1875 throat swab specimens were collected from 187 COVID-19 confirmed patients who detected SARS-CoV-2 RNA for equal to or more than 5 times. Each patient test SARS-CoV-2 RNA for 10 times on average and the total positive rate was 40.65% (763/1877) (Supplementary Table S1). The results of sequential SARS-CoV-2 RNA detection results for each patient within day 60 were shown in Supplementary Figure S1. The median positive conversion time for SARS-CoV-2 RNA (defined as the first positive time point of RNA tests) was day 7 (IQR, 3–11). There were 160 patients (85.56%, 160/187) received positive results at the first time of RNA test, and the other 27 patients (14.44%, 27/187) underwent multiple RNA tests until positive results were obtained.

**Figure 1.**
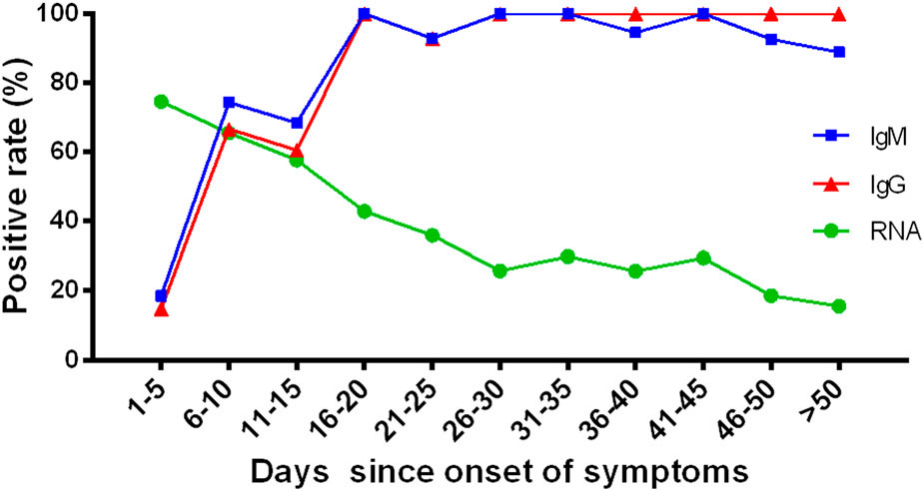
The positive rates of RNA and antibodies among COVID-19 patients in different time durations since symptoms onset. The positive rate of RNA was analysed in 1875 throat swab specimens from 187 COVID-19 confirmed patients. The positive rate of antibodies was analysed in 409 serum samples from 409 COVID-19 confirmed patients.

Considering the disease stage may affect the detection of viral RNA, we analysed the positive rate of throat swabs for SARS-CoV-2 RNA in different stages (Figure 1 and Supplementary Table S1). It was clearly observed that the positive rate of RNA was the highest in the early stage (day 1–5) and gradually decreased as the disease duration was prolonged. The RNA positive rate decreased from 74.59% in the early stage (day 1–5) to 15.67% in the advanced stage (day > 50 since onset).

### Positive conversion of antibodies and RNA against SARS-CoV-2 in patients with COVID-19

**Figure 2.**
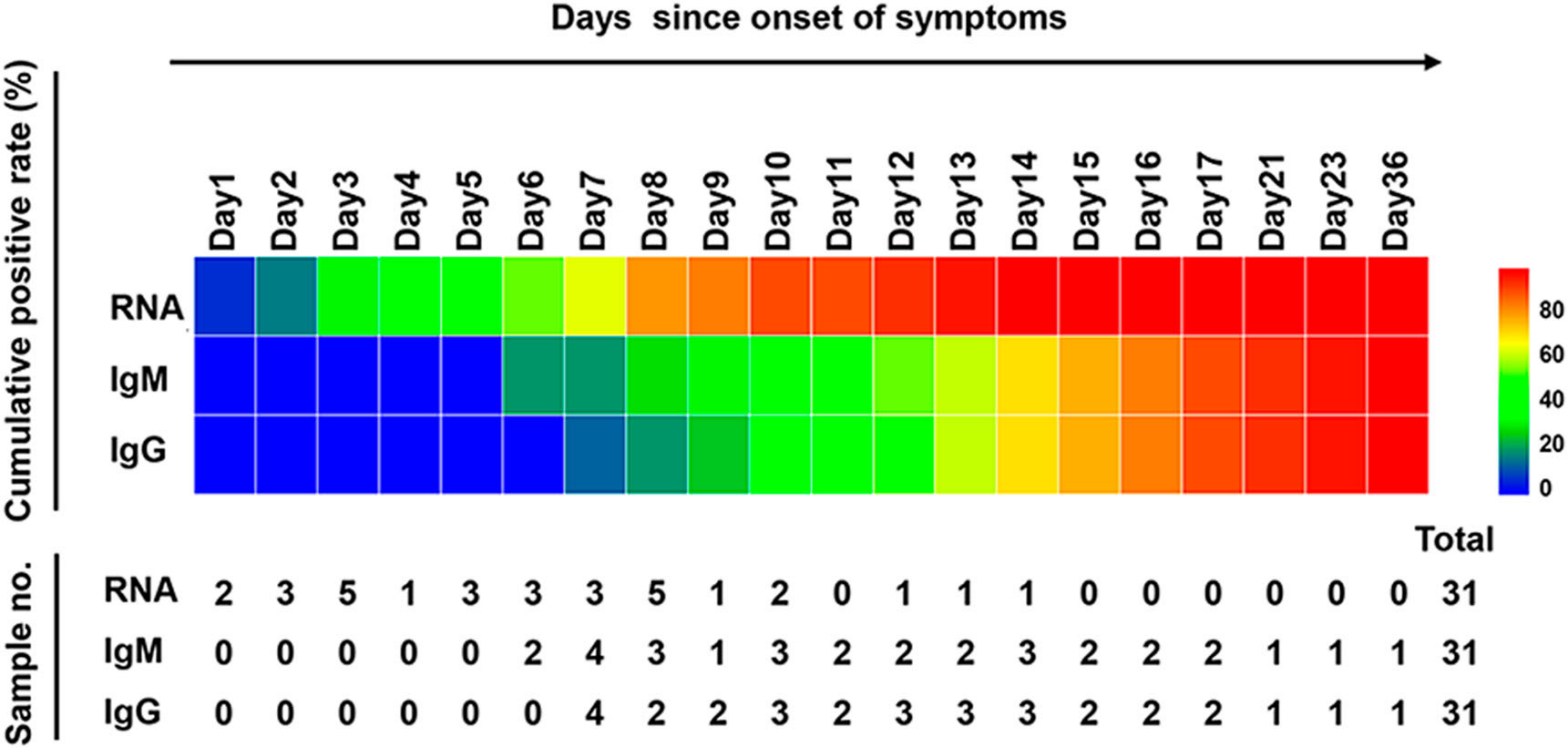
Positive conversion of RNA and antibodies against SARS-CoV-2 in COVID-19 patients. Cumulative positive rates of patients with COVID-19 for RNA and antibody (IgM, IgG) were displayed by courses of the disease. The scale on the right of the figure shows the positive rate level indicated with different colours, the blue corresponds to a low positive rate and the red to a high positive rate. The lower table presents the number of samples that tested positive at each time point.

A total of 162 serial serum samples were collected from 31 COVID-19 patients during the hospitalization period. All the serum specimens of these patients were collected and tested for equal to or more than three times. And each patient tested for antibodies against SARS-CoV-2 for 5.23 times on average. The median time of seroconversion for IgM and IgG were both the day 12 (IQR, 10–15). The cumulative seropositive rate reached 54.84% and 51.61% on the 12th day for IgM and IgG, respectively (Figure 2). Moreover, the cumulative seropositive rate reached 100% on the 36th-day for both IgM and IgG (Figure 2). The seroconversion was closely and sequentially appeared for IgM and then IgG.

**Figure 3.**
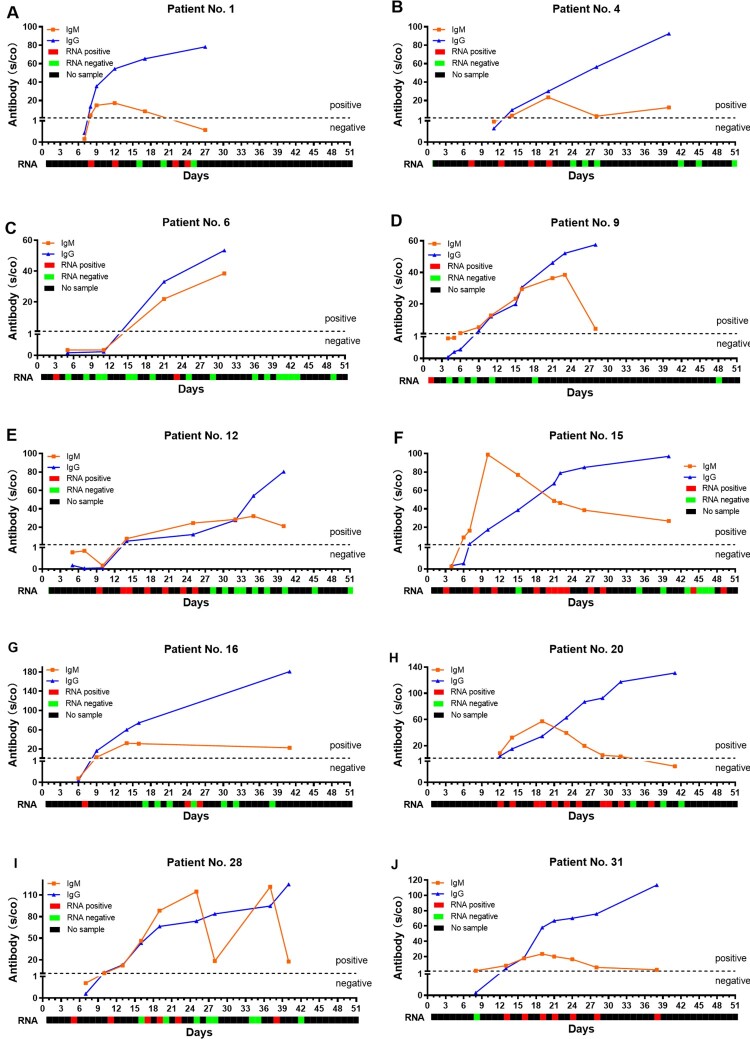
The longitudinal changes of antibody and RNA in 10 representative patients with COVID-19. The red solid square represents the positive result of RNA, the green solid square represents the negative result of RNA, and the black solid square represents no sample was collected.

In order to know the changes for antibody and RNA against the SARS-CoV-2 in the same patient, we further analysed the RNA result of these 31 patients before day 51. The cumulative RNA positive rate was 64.52% before the 7th day, and it reached 100% on the 14th-day (Figure 2). The longitudinal changes of antibody and RNA in 10 representative patients were presented in Figure 3, and the others were displayed in Supplementary Figure S2. The first positive time point of RNA tests appeared earlier than that of IgM in 28 of 31 patients (28/31, 90.32%), except for patient No.1, No.20 and No.31 (at the same day). When it comes to IgG, the seroconversion time of 29 cases (29/31, 93.55%) were later than the positive conversion time of RNA, and the rest (2/31, 6.45%, patient No.1 and No.20) were the same as RNA. It should be noted that the risings of antibodies were not always accompanied by RNA clearance, especially the patient No. 31 (Figure 3(J)).

### The sensitivity and specificity for serological methods

We enrolled 409 cases (217 males vs. 192 females) with COVID-19 and 389 controls (224 males vs. 165 females) so as to explore the sensitivity and specificity for serological methods. And the median age was 60 years (IQR, 49-69) and 45 years (IQR, 29-61) for patient with and without COVID-19, respectively (Table 1).

**Table 1.** Summary results for IgM and IgG detection in 798 plasma samples from patients with/without COVID-19.

Variables		COVID-19 patients, *n* (%)	Non-COVID-19 patients, *n* (%)	Sensitivity (%, 95%CI)	Specificity (%, 95%CI)	Kappa
Total		409	389			
Age	median (IQR)	60 (49–69)	45 (29–61)			
Sex						
	Male	217 (53.06)	224 (57.58)			
	Female	192 (46.94)	165 (42.42)			
IgM				87.04 (83.77–90.31)	96.92 (95.19–98.64)	0.84
	Positive	356 (87.04)	12 (1.05)			
	Negative	53 (12.96)	377 (98.95)			
IgG				87.04 (83.77–90.31)	96.92 (95.19–98.64)	0.84
	Positive	356 (87.04)	12 (1.05)			
	Negative	53 (12.96)	377 (98.95)			
IgM/IgG				88.75 (85.68–91.83)	96.14 (94.22–98.07)	0.85
	Positive	363 (88.75)	15 (1.84)			
	Negative	46 (11.25)	374 (98.16)			

Abbreviation: IQR, interquartile range; CI, confidence interval.

IgM/IgG: at least one positive result detected by IgM and IgG.

The time between the symptoms onset and the collection of serum samples ranged from 1 to 87 days.

The IgM and IgG antibodies were detected positive as early as on the day-3 since onset of the symptom. The sensitivity and specificity for IgM to distinguish the COVID-19 patients and non- COVID-19 patients was 87.04% (95% CI, 83.77–90.31%) and 96.92% (95% CI, 95.19–98.64%), respectively. As for IgG, it had the same sensitivity and specificity as IgM. Furthermore, the serological methods showed a high consistent with the results of SARS-CoV-2 RT-qPCR testing (kappa = 0.84, *p* < 0.0001, both IgM and IgG). Noteworthy, combining the results of IgM and IgG (patients with either IgM or IgG positive) would increase the sensitivity to 88.75% (95% CI, 85.68–91.83%), and the consistency with RNA to 0.85 (kappa = 0.85, *p* < 0.0001). Moreover, 15 controls (negative SARS-CoV-2 RT-qPCR testing) had positive results for IgM or IgG, all these cases with respiratory symptoms, which indicated that these cases may be missed by SARS-CoV-2 RT-qPCR testing or false positive results. However, there were 53 samples showed seronegative for both IgM and IgG, possibly due to that most samples (39/53, 73.58% for IgM; 45/53, 84.91% for IgG) involved were collected at the early stage of illness (IgM: 39 earlier than day-12, 5 on day-13 to day-15, the other 9 were later than day-20; IgG: 45 earlier than day-12, 6 on day-13 to day-15, the other 2 were later than day-20).

### The diagnostic performance of serological methods for patients in different stages since symptoms onset

To study the performance of antibodies in different stages of COVID-19 patients, we divided the patients into eleven groups based on the number of days from symptoms onset to serum collection (1–5, 6–10, 11–15, 16–20, 21–25, 26–30, 31–35, 36–40, 41–45, 46–50 and >50). The positive rates of IgM and IgG in different groups are shown in Supplementary Table S2. There was a clear increase in positive rates for both IgM and IgG (Figure 1). The positive rates of IgM and IgG were both low at the day 1–5 (18.52% for IgM, 14.81% for IgG), and significantly increased at day 16–20 (100% for both IgM and IgG). And the positive rate of IgM was higher than that of IgG at the day earlier than 15 (Figure 1). Moreover, the IgM/IgG testing had a crossing with the RT-qPCR method at about 10 days. There was a higher positive rate for RNA than antibody at and before the day 10 since onset of symptom, and reversed after 10 days.

### The logistic regression analysis identified influence factors for seroconversion in patients with COVID-19

**Figure 4.**
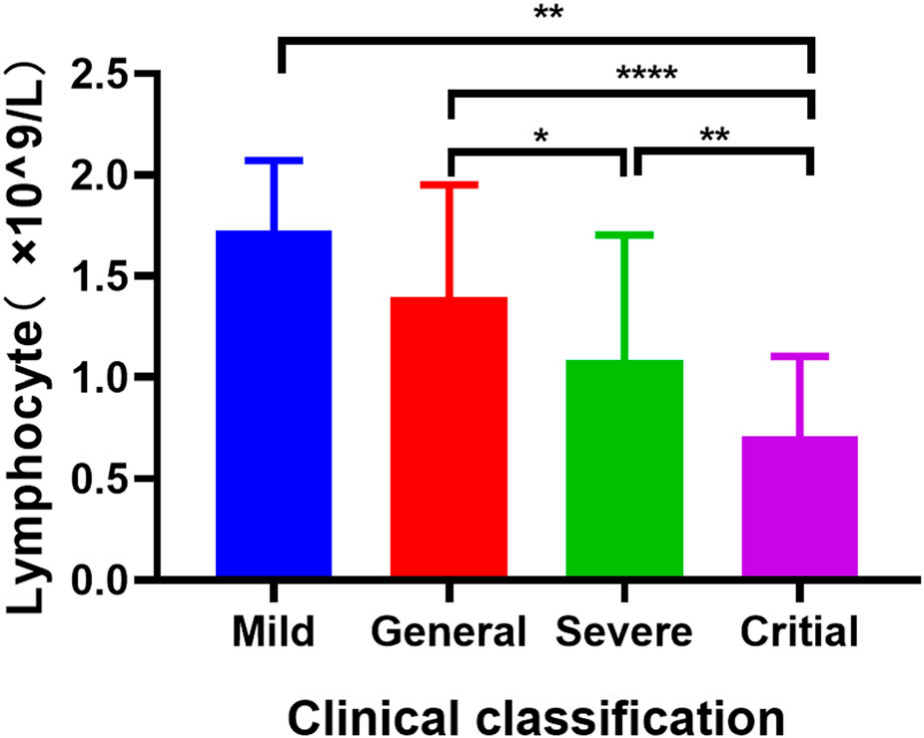
Lymphocyte counts in different subtypes of COVID-19 patients. Independent-samples t test was used to comparison the variables in different groups. * *p* < 0.05, ** *p* < 0.01, **** *p* < 0.0001.

There were 163 confirmed COVID-19 patients with complete clinical data were selected to compare the differences in the baseline demographic, clinical and biochemical characteristics of IgM (or IgG) positive and negative patients (Table 2). Of these patients, 130 (79.75%) and 127 (77.91%) patients tested positive for IgM and IgG antibodies, respectively. Female patients with COVID-19 were more prone to have negative results for IgG (*p *= 0.0148). Compared with the antibody positive patients, patients with negative antibody tests had lower lymphocyte counts (*p *= 0.0005 for IgM, *p *= 0.0046 for IgG) and a higher proportion of critical degree patients (*p *= 0.0014 for IgM, *p *= 0.0190 for IgG), while as disease severity increasing the lymphocyte counts was gradually decreased (Figure 4). Besides, individuals with short SARS-CoV-2 RNA positive conversion time were more likely to have negative antibodies test results (*p *= 0.0228 for IgM, *p *= 0.0022 for IgG). Noteworthy, the comorbidities, such as hypertension (*p *= 0.0239) and cardiovascular-cerebrovascular diseases (*p *= 0.0190) might also correlate with negative results in IgM test.

**Table 2.** Baseline characteristics of IgM/IgG positive/negative patients with COVID-2019.

Variables	IgM (*n* = 163)		*p*	IgG (*n* = 163)		
	Positive	Negative		Positive	Negative	*p*
No.	130	33		127	36	
Age, year	53.86 ± 14.98	57.52 ± 15.31	0.2147^a^	54.02 ± 14.97	56.67 ± 15.49	0.3533^a^
Gender						
Male, *n* (%)	75 (57.69%)	13 (39.39%)	0.0596^b^	75 (59.06%)	13 (36.11%)	**0.0148**^b^
Female, *n* (%)	55 (42.31%)	20 (60.61%)		52 (40.94%)	23 (63.89%)	
Comorbidities						
Diabetes, *n* (%)	22 (16.92%)	5 (15.15%)	0.8069^b^	22 (17.32%)	5 (13.89%)	0.6247^b^
Hypertension, *n* (%)	33 (25.38%)	15 (45.45%)	**0.0239**^b^	33 (25.98%)	14 (38.89%)	0.1314^b^
Cardia-cerebrovascular disease, *n* (%)	20 (15.38%)	11 (33.33%)	**0.0190**^b^	21 (16.54%)	10 (27.78%)	0.1292^b^
Respiratory diseases, *n* (%)	5 (3.85%)	3 (9.09%)	0.2130^b^	4 (3.15%)	4 (11.11%)	0.0724^b^
Tumor, *n* (%)	3 (2.31%)	2 (6.06%)	0.2665^b^	3 (2.36%)	2 (5.56%)	0.3051^b^
Clinical classification						
Mild, *n* (%)	2 (1.54%)	0 (0)	N/A	2 (1.57%)	0 (0)	N/A
General, *n* (%)	86 (66.15%)	18 (54.55%)	0.2153^b^	82 (64.57%)	22 (61.11%)	0.7033^b^
Severe, *n* (%)	23 (17.69%)	2 (6.06%)	0.1121^b^	23 (18.11%)	2 (5.56%)	0.0650^b^
Critical, *n* (%)	19 (14.62%)	13 (39.39%)	**0.0014**^b^	20 (15.75%)	12 (33.33%)	**0.0190**^b^
Positive conversion time for SARS-CoV-2 RNA	7.50 (4.00–13.00)	5.00 (3.00–7.50)	**0.0228**^c^	8.00 (4.00–13.00)	5.00 (3.00–7.00)	**0.0022**^c^
Clinical laboratory test						
WBC, 10^^9/^L	5.59 (4.46–7.35)	4.86 (3.44–8.58)	0.4902^c^	5.63 (4.53–7.42)	4.82 (3.40–8.32)	0.3250 ^c^
Neutrophil, 10^^9/^L	3.47 (2.40–5.17)	3.47 (1.85–7.41)	0.8751^c^	3.56 (2.44–5.24)	3.17 (1.93–7.08)	0.5982^c^
Lymphocyte, 10^^9/^L	1.30 ± 0.61	0.90 ± 0.43	**0.0005**^a^	1.29 ± 0.61	0.97 ± 0.49	**0.0046**^a^
Monocyte, 10^^9/^L	0.45 (0.33–0.64)	0.42 (0.31–0.56)	0.3646^c^	0.44 (0.34–0.64)	0.42 (0.31–0.56)	0.4724^c^
RDW, (%)	13.20 (12.68–13.70)	13.10 (12.55–13.95)	0.9975^c^	13.20 (12.70–13.70)	13.05 (12.43–13.90)	0.7160^c^
Glucose, mmol/L	5.48 (4.86–6.85)	5.98 (5.12–6.99)	0.2702^c^	5.49 (4.86–7.02)	5.82 (5.09–6.77)	0.3930^c^
ALT, U/L	27.00 (18.75–50.25)	24.00 (14.50–41.50)	0.2106^c^	28.00 (20.00–50.25)	20.50 (14.00–39.50)	**0.0274**^c^
AST, U/L	25.50 (19.00–30.00)	26.00 (19.50–51.00)	0.5849^c^	26.00 (19.00–40.00)	25.50 (16.50–47.75)	0.5491^c^
BUN, mmol/L	4.56 (3.71–5.66)	5.21 (3.49–8.36)	0.1119^c^	4.55 (3.72–5.65)	5.15 (3.53–7.91)	0.1291^c^
CRP, mg/L	8.45 (2.08–46.15)	15.23 (3.03–43.90)	0.5316^c^	8.90 (2.00–48.70)	10.90 (2.95–44.15)	0.6416^c^

Data was displayed as mean ± SD, median (IQR, interquartile range), or *n* (%).

^a^two-tailed Student’s *t* test

^b^*χ*² test

^c^non-parametric test (Mann–Whitney *U* test)

Positive conversion time for SARS-CoV-2 RNA defined as the first positive time point of RNA tests.

*p* < 0.05 was considered statistically significant (in bold).

Abbreviation: WBC, white blood cell; ALT, alanine transaminase; AST, aspartate transaminase; BUN, blood urea nitrogen; CRP, C-reactive protein; RDW, red blood cell distribution width; N/A, not applicable.

Univariate and multivariate binary logistic analysis were used to analyse the influencing factors of antibody testing (Table 3). Univariate logistic analysis revealed that lymphocyte counts (*p *= 0.001 for IgM, *p *= 0.006 for IgG) and SARS-CoV-2 RNA positive conversion time (*p *= 0.014 for IgM, *p *= 0.003 for IgG) were positively associated with antibody positive results, while, the critical patients had a higher negative rate of IgM (*p *= 0.002) and IgG (*p *= 0.022). Additionally, cardiovascular and cerebrovascular diseases negatively associated with IgM positive results (*p *= 0.022), and the male (*p *= 0.017) and serum ALT level (*p *= 0.029) positively associated with IgG positive results. Multivariate logistic regression models indicated that the lymphocyte counts (*p *= 0.017 for IgM, *p *= 0.043 for IgG) and SARS-CoV-2 RNA positive conversion time (*p *= 0.014 for IgM, *p *= 0.003 for IgG) were the independent influence factors for antibody positive results after the adjustment for demographic and clinical characteristics. In addition, gender was also an independent factor that affecting IgG detection (*p *= 0.032).

**Table 3.** Univariate and multivariate binary logistic analysis of influencing factors of IgM / IgG result judgment.

Variables	IgM positive/negative (*n* = 163)				IgG positive/negative (*n* = 163)			
	Univariate		Multivariate		Univariate		Multivariate	
	OR (95%CI)	*p*	OR (95%CI)	*p*	OR (95%CI)	*p*	OR (95%CI)	*p*
Age	0.98 (0.96–1.01)	0.215	1.00 (0.97–1.04)	0.987	0.99 (0.96–1.01)	0.351	1.00 (0.96–1.03)	0.804
Gender	2.10 (0.96–4.58)	0.063	2.61 (0.92–7.42)	0.072	2.55 (1.19–5.49)	**0**.**017**	3.05 (1.10–8.48)	**0**.**032**
Comorbidities						** **		** **
Diabetes	1.14 (0.40–3.28)	0.807	1.53 (0.37–6.30)	0.554	1.30 (0.45–3.71)	0.625	1.67 (0.40–6.96)	0.481
Hypertension	0.46 (0.21–1.02)	0.057	0.45 (0.14–1.42)	0.174	0.55 (0.25–1.20)	0.134	0.46 (0.14–1.46)	0.186
Cardia-cerebrovascular disease	0.36 (0.15–0.87)	**0**.**022**	0.65 (0.18–2.42)	0.523	0.52 (0.22–1.23)	0.134	1.04 (0.26–4.11)	0.952
Respiratory diseases	0.40 (0.91–1.77)	0.227	0.32 (0.05–1.91)	0.209	0.26 (0.06–1.10)	0.067	0.15 (0.03–0.90)	0.038
Tumor	0.37 (0.60–2.29)	0.282	0.19 (0.02–2.04)	0.172	0.41 (0.07–2.56)	0.341	0.20 (0.02–2.23)	0.192
Clinical classification								
Mild	N/A		N/A		N/A		N/A	
General	0.61 (0.28–1.33)	0.218	1.0 (Ref.)		0.86 (0.40–1.85)	0.703	1.0 (Ref.)	
Severe	0.30 (0.07–1.34)	0.116	2.90 (0.49–17.09)	0.239	0.27 (0.60–1.19)	0.083	3.34 (0.55–20.37)	0.191
Critical	0.26 (0.11–0.62)	**0**.**002**	0.35 (0.07–1.71)	0.197	0.37 (0.16–0.87)	**0**.**022**	0.35 (0.07–1.78)	0.206
Positive conversion time for SARS-CoV-2 RNA	1.11 (1.02–1.21)	**0**.**014**	1.16 (1.03–1.31)	**0**.**014**	1.14 (1.05–1.25)	**0**.**003**	1.21 (1.07–1.38)	**0**.**003**
Clinical laboratory test								
WBC, 10^^9/^L	1.01 (0.91–1.11)	0.900			1.01 (0.92–1.12)	0.793		
Neutrophil, 10^^9/^L	0.98 (0.89–1.07)	0.644			0.99 (0.90–1.09)	0.822		
Lymphocyte, 10^^9/^L	3.91 (1.73–8.821)	**0**.**001**	3.48(1.25–9.71)	**0**.**017**	2.77 (1.34–5.74)	**0**.**006**	2.68 (1.03–6.99)	**0**.**043**
Monocyte, 10^^9/^L	1.68 (0.30–9.47)	0.559			1.30 (0.25–6.69)	0.755		
RDW, (%)	0.82 (0.66–1.03)	0.088			0.85 (0.68–1.06)	0.157		
Glucose, mmol/L	0.96 (0.84–1.10)	0.568			0.99 (0.86–1.13)	0.843		
ALT, U/L	1.02 (1.00–1.03)	0.087	1.02( 0.99–1.04)	0.220	1.02 (1.00–1.04)	**0**.**029**	1.02 (0.99–1.04)	0.151
AST, U/L	1.00 (0.99–1.01)	0.929			1.01 (0.99–1.02)	0.501		
BUN, mmol/L	0.95 (0.90–1.01)	0.122			0.98 (0.92–1.04)	0.406		
CRP, mg/L	1.00 (1.00–1.01)	0.485			1.00 (1.00–1.01)	0.375		

Abbreviation: WBC, white blood cell; ALT, alanine transaminase; AST, aspartate transaminase; BUN, blood urea nitrogen; CRP, C-reactive protein; RDW, red blood cell distribution width; IL-6, interleukin-6; N/A, not applicable; Ref, reference.

*p* < 0.05 was considered statistically significant (in bold).

## Discussion

In the present study, we reviewed the positive conversion for RNA and antibody (IgM and IgG) in patients with COVID-19 and recommended the suitable time duration for positive test results. The median positive conversion time for RNA was the 7th day, while the median seroconversion time for IgM and IgG were both the 12nd days since onset of symptoms. Though the single detection for swabs RNA or antibodies was not enough for helping the diagnosis of COVID-19, combining detection assays at different times could maximize the positive rate. The results suggested that antibody tests could be an important supplement to RNA detection during the illness course, but some special patients with decreased lymphocytes could remain negative of antibody detection results.

In this study, we analysed the consecutive throat swabs specimens from 187 COVID-19 patients. The median positive conversion time for SARS-CoV-2 RNA was day 7 (IQR, 3–11), which was consist with Chen et al. [10] reported day 6.5. The total positive rate was 40.65%, which was broadly in line with previously reported 38% [5]. However, the RT-qPCR showed a high positive rate (74.59%) in the early stage (day 1–5), and gradually decreased as the disease duration was prolonged (less than 20% at day > 45, Figure 1, Supplementary Table S1). Notably, there were 27 patients (14.44%, 27/187) underwent multiple negative nucleic acid tests before confirmed diagnosis were made. This indicated that in addition to multiple tests for RNA with the same patient, other detection techniques are needed in the supplement to the current diagnostic shortages for RNA testing, especially at the late stage.

The results of IgM and IgG from serial serum samples suggested that 100% of the patients had antibody responses to SARS-CoV-2 during the course of disease. The median day of seroconversion for both IgG and IgM were 12 days since the symptom onset, which was to a certain extent in accordance with a previous publication. As Long et al. [11] found that the median day of seroconversion for both IgG and IgM was 13 days since symptom onset; while Zhao et al. [12] reported the median seroconversion day of IgM and IgG was day-12 and day-14, respectively. However, the reported median day of seroconversion was variable and ranged from 5 to 14 days [11–14], which required consideration of the sensitivity of the assay, the type of antigen, sample size and the duration of follow-up for the cohort [14]. Of note, there was one patient (No. 13, Figure S2) obtained the positive results of both IgM and IgG at day 36. The late seroconversion of antibody had also been reported in SARS-CoV. As Chen et al. [15] reported that the antibody of SARS-CoV might be positive as early as 8–10 days since the onset of illness and often by day 14, but sometimes a positive result was not achieved until 28 days since illness onset. In addition to the error caused by the lack of intermediate serum samples, impaired immune function might also contribute to the blunted responses against SARS-CoV-2 antigens [16].

Moreover, our results from 409 patients with COVID-19 showed that the positive rates of IgM and IgG were both low in early stage (Figure 1 and Supplementary Table S2), and gradually increased with the prolongation of disease courses, which consistent with the previous study [13,17]. However, the positive rate of RNA was the highest in the early stage and gradually decreased as the disease duration was prolonged. Moreover, there was a crossing for the RT-qPCR method and IgM/IgG testing at about 10 days, which was later than Guo, et al reported 5.5 days [13]. Anyway, the serological methods of IgM or IgG could be an important supplement to RNA detection, especially during the later illness course (such as > 10 days), and RNA detection can supplement the low sensitivity of antibody detection in the early stages of illness course. Combined RNA and antibody detection could potentially improve diagnostic efficiency. However, there were 53 patients confirmed by RT-qPCR had negative results of IgM or IgG. This may because the majority of these serum samples (73.58% for IgM, 84.91% for IgG) were collected at an earlier stage (≤ day 12), the specific immune response had not yet occurred or the antibody titres were too low to detect [9,13].

Multivariate logistic regression indicated that decreased lymphocyte counts were the independent factors leading to negative results of IgM and IgG antibody tests. As lymphocytes played an important role in producing antibodies, and reduced lymphocytes were common clinical features of COVID-19 patients, which suggested the impaired immune system of SARS-CoV-2 infected patients [18]. Moreover, decreased lymphocyte counts were associated with the disease severity, prognosis, and mortality of hospitalized COVID-19 patients [11,19–21]. Consistent with previous researches, we also found that as the severity of the disease increased, lymphocytes showed a significant gradual decline in COVID-19 patients. Moreover, Shen et al. [22] and Kong et al. [23] reported that delayed antibody responses among severe COVID-19 patients. So we speculate that the decrease in lymphocytes of critically patients may lead to weak antibody responses or even negative antibody results in COVID-19 patients. Inconsistently, Zhao et al. [12] reported that a high antibody titre was a risk factor of critical illness, this might due to the antibodies used were different (we used antibodies against SARS-CoV-2 spike protein). As Sun et al. [24] reported that S-IgG against SARS-CoV-2 spike protein was significantly lower in ICU patients than in non-ICU patients, but the N-IgG against SARS-CoV-2 nucleocapsid protein showed the opposite result. Interestingly, the positive conversion time for SARS-CoV-2 RNA in antibody negative patients was significantly shorter than that in positive patients, and the short time for the positive detection might indicate the higher virus load [25,26]. Similarly, the levels of antibodies were inversely correlated with viral loads for MERS [27]. Furthermore, we found that gender was also an independent factor that affecting IgG detection, this may be due to the sex-specific immunobiological differences [28], but the exact mechanism was unclear. These data suggested that beside the course of disease, virus load as well as host immunology status could contribute toward the antibody response to the virus.

It should be noted that there were some limitations in this study. Firstly, this was a retrospective study which inherited all retrospective analyses limitation such as integrity of data, leading to incomplete overlap of the patients in different part. Secondly, qualitative RNA tests of patients were only based on upper respiratory tract specimens, lack of quantitative detection, and relatively low positive rate of SARS-CoV-2 RNA detection in throat swabs [25]. Thirdly, the sample size of serial serum analysis was small. Finally, the specificity analysis was inadequate in this study due to there was no real healthy controls.

In conclusion, this study indicated that the antibody response to SARS-CoV-2 could aid in the diagnosis of COVID-19. The RNA testing displayed a high positive rate in the early stage, especially day 1–5, and the antibody methods showed a higher positive rate than that of RNA after 10 days since symptoms onset. We suggested to select different detection methods in different stages of the disease in order to improve the positive detection rate of patients with COVID-19. From the perspective of health economics, RNA detection in the early stage (1–5 day), combination of the RNA and antibody detection in the middle stage (6–45 day), and antibody detection in the advanced stage (> 45 day) could help to get the supporting result for the rapid diagnosis of COVID-19 (Table 4). And the understanding on the dynamic changes of RNA and antibody results, will help explaining the false negative results of RNA detection or antibody detection in the patients in different disease courses. Furthermore, this study was the first report about the influence factors of decreased lymphocytes and short positive conversion time on conversion of IgM and IgG against SARS-CoV-2.

**Table 4.** Recommendations for laboratory testing of COVID-19 at different disease course.

General days since symptom onset	RNA	IgM	IgG
1–5	++	−	−
6–10	++	+	+
11–45	+	++	++
> 45	-	++	++

++: strong recommendation.

+: general recommendation.

−: not recommend.

## Supplementary Material

Supplementary_Figure_S2.tif

Supplementary_Figure_S1.tif

Supplementary_Table_S2.doc

Supplementary_Table_S1.doc

Supplementary_information.doc
