# Supplementary figures and images for: Dynamic changes of throat swabs RNA and serum antibodies for SARS-CoV-2 and their diagnostic performances in patients with COVID-19
Source: Emerg Microbes Infect. 2020 Sep 17;9(1):1974–83. doi: 10.1080/22221751.2020.1810133 (PMC7534196; doi:10.1080/22221751.2020.1810133)

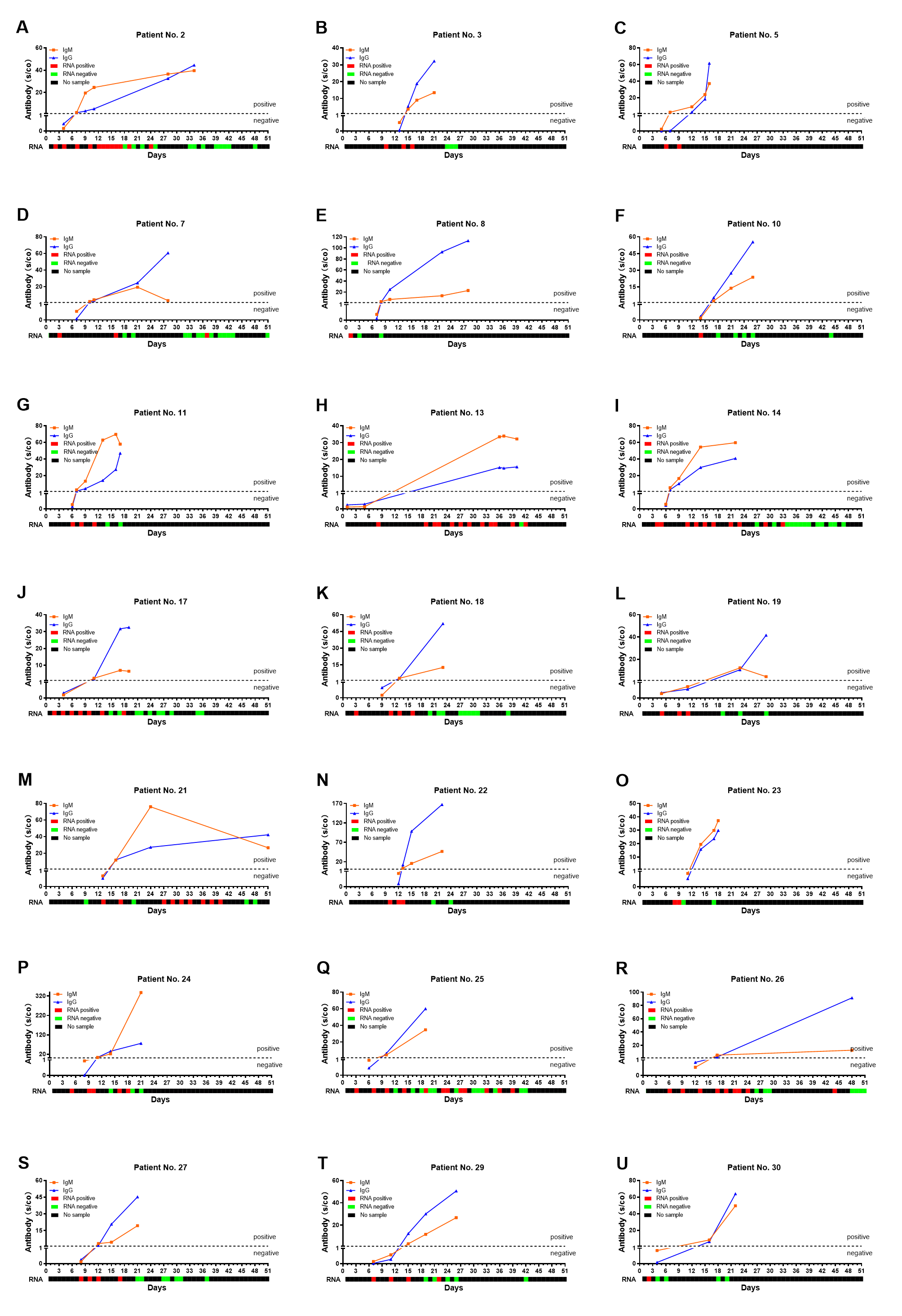

Supplement: Supplementary_Figure_S2.tif [file TEMI_A_1810133_SM3772.tif]

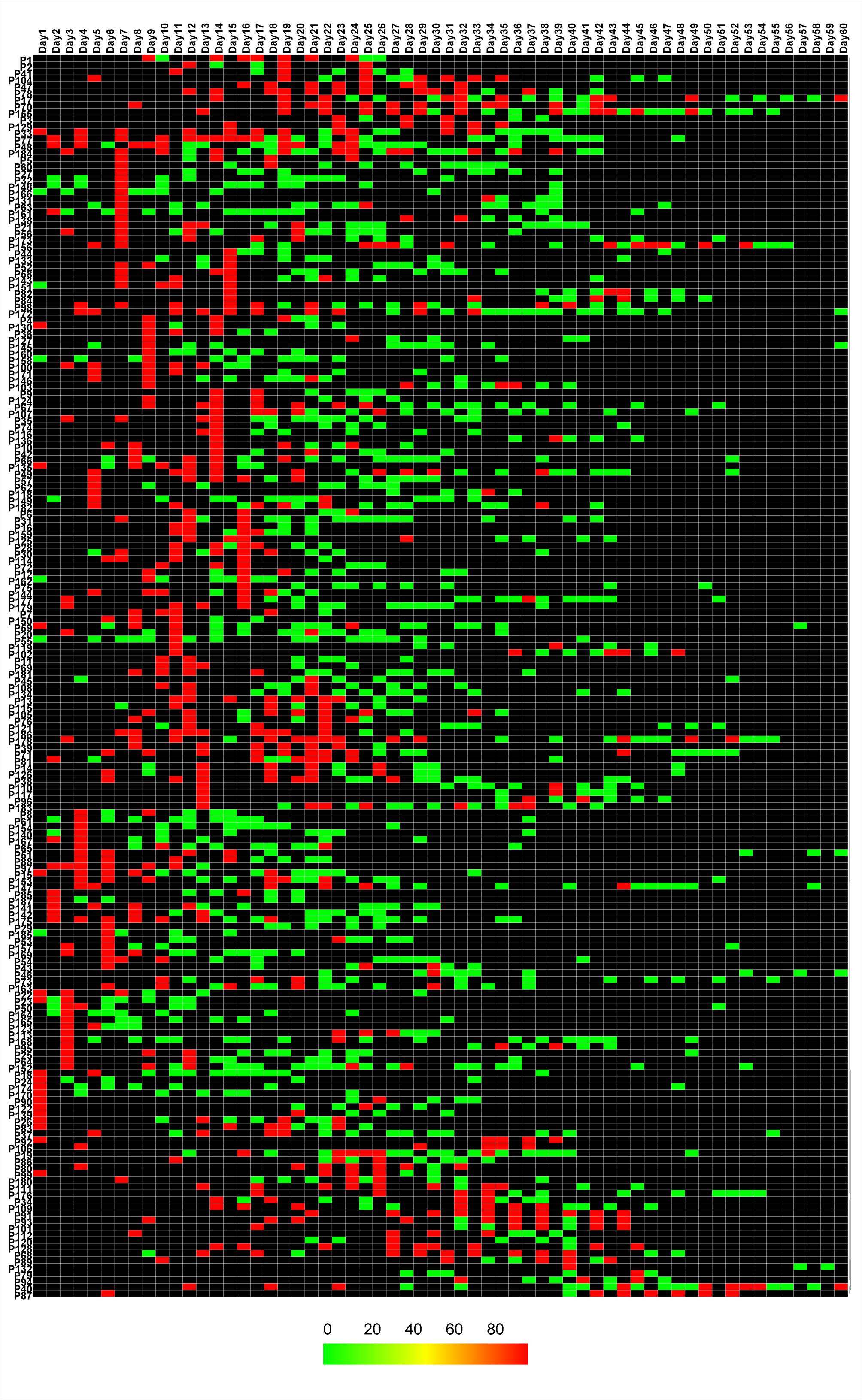

Supplement: Supplementary_Figure_S1.tif [file TEMI_A_1810133_SM3771.tif]
