# Supplementary material for: Dynamic changes of throat swabs RNA and serum antibodies for SARS-CoV-2 and their diagnostic performances in patients with COVID-19
Source: Emerg Microbes Infect. 2020 Sep 17;9(1):1974–83. doi: 10.1080/22221751.2020.1810133 (PMC7534196; doi:10.1080/22221751.2020.1810133)
Supplement: Supplementary_Table_S2.doc [file TEMI_A_1810133_SM3770.doc]

**Supplementary Table S2.** Positive rate for detection of IgM and IgG in plasma samples of patients with COVID-19 at different stages since symptoms onset.

| Days | No. of serum samples | IgM | |  | IgG | |
| --- | --- | --- | --- | --- | --- | --- |
|  |  | Positive no. | % |  | Positive no. | % |
| 1-5 | 27 | 5 | 18.52 |  | 4 | 14.81 |
| 6-10 | 39 | 29 | 74.36 |  | 26 | 66.67 |
| 11-15 | 38 | 26 | 68.42 |  | 23 | 60.53 |
| 16-20 | 32 | 32 | 100.00 |  | 32 | 100.00 |
| 21-25 | 28 | 26 | 92.86 |  | 26 | 92.86 |
| 26-30 | 48 | 48 | 100.00 |  | 48 | 100.00 |
| 31-35 | 52 | 52 | 100.00 |  | 52 | 100.00 |
| 36-40 | 55 | 52 | 94.55 |  | 55 | 100.00 |
| 41-45 | 45 | 45 | 100.00 |  | 45 | 100.00 |
| 46-50 | 27 | 25 | 92.59 |  | 27 | 100.00 |
| >50 | 18 | 16 | 88.89 |  | 18 | 100.00 |
| Total | 409 | 356 | 87.04 |  | 356 | 87.04 |
