# Supplementary material for: Dynamic changes of throat swabs RNA and serum antibodies for SARS-CoV-2 and their diagnostic performances in patients with COVID-19
Source: Emerg Microbes Infect. 2020 Sep 17;9(1):1974–83. doi: 10.1080/22221751.2020.1810133 (PMC7534196; doi:10.1080/22221751.2020.1810133)
Supplement: Supplementary_Table_S1.doc [file TEMI_A_1810133_SM3769.doc]

**Supplementary Table S1.** The positive rates of 1877 throat swabs specimens for SARS-CoV-2 RNA in different disease stages since symptoms onset.

| Days | No. of throat swab samples | RNA | | |
| --- | --- | --- | --- | --- |
|  |  | No. | Positive (%) |  |
| 1-5 | 122 | 91 | 74.59 |  |
| 6-10 | 177 | 116 | 65.54 |  |
| 11-15 | 239 | 138 | 57.74 |  |
| 16-20 | 268 | 115 | 42.91 |  |
| 21-25 | 278 | 100 | 35.97 |  |
| 26-30 | 217 | 56 | 25.81 |  |
| 31-35 | 174 | 52 | 29.89 |  |
| 36-40 | 148 | 38 | 25.68 |  |
| 41-45 | 112 | 33 | 29.46 |  |
| 46-50 | 59 | 11 | 18.64 |  |
| >50 | 83 | 13 | 15.67 |  |
| Total | 1877 | 763 | 40.65 |  |
