# Supplementary material for: Dynamic changes of throat swabs RNA and serum antibodies for SARS-CoV-2 and their diagnostic performances in patients with COVID-19
Source: Emerg Microbes Infect. 2020 Sep 17;9(1):1974–83. doi: 10.1080/22221751.2020.1810133 (PMC7534196; doi:10.1080/22221751.2020.1810133)
Supplement: Supplementary_information.doc [file TEMI_A_1810133_SM3768.doc]

**Supplementary information**

**Supplementary Table S1.** The positive rates of 1877 throat swabs specimens for SARS-CoV-2 RNA in different disease stages since symptoms onset.

**Supplementary Table S2.** Positive rate for detection of IgM and IgG in plasma samples of patients with COVID-19 at different stages since symptoms onset.

**Supplementary Figure S1**. The results of sequential SARS-CoV-2 RNA detection results from 187 COVID-19 patients within day 60. Each row represents the results of one patient in different days since symptom onset. The red solid square represents the positive result of RNA, the green solid square represents the negative result of RNA, and the black solid square represents no sample was collected.

**Supplementary Figure S2**. The longitudinal changes of antibody and RNA in 21 patients with COVID-19. The red solid square represents the positive result of RNA, the green solid square represents the negative result of RNA, and the black solid square represents no sample was collected.
